# ADJUSTMENT TO AGING AMONG LBGT+ OLDER ADULTS

**DOI:** 10.1093/geroni/igac059.1903

**Published:** 2022-12-20

**Authors:** Sofia von Humboldt, Francis Carneiro, Isabel Leal

**Affiliations:** ISPA - Instituto Universitário, Lisbon, Lisboa, Portugal; ISPA - Instituto Universitário, Lisbon, Lisboa, Portugal; ISPA - Instituto Universitário, Lisbon, Lisboa, Portugal

## Abstract

Intervention programs that highlight predictors of adjustment to aging (AtA)(1) for minority older lesbian, gay and bisexual (LGB) populations are scarce(2).The aim of this preliminary study is to build a structural model to explore whether socio-demographic, health and lifestyle-related variables, are correlates of AtA in a group of LGB older adults(3).The sample comprised 287 LGB older adults aged 75 years old and older.Convenience sampling was used to gather questionnaire data.Measures encompassed the Adjustment to Aging Scale, the Satisfaction with Life Scale, demographics and lifestyle and health-related characteristics.Structural equation modeling was used to explore a structural model of the self-reported AtA, comprising all the above variables.The structural model indicated the following significant correlates: perceived health(β=0.456; p< 0.001), leisure(β=0.378; p< 0.001), income(β=0.302; p< 0.001), education(β=0.299; p=0.009), spirituality(β=0.189; p< 0.001), sex(β=0.156; p< 0.001), physical activity(β=0.142; p< 0 .001), satisfaction with life(β=0.126; p< 0.001), and marital status(β=0.114; p=0.008).The variables explain respectively 76.4% of the variability of AtA. These outcomes suggest that policy making and community interventions with LGB older adults may benefit of including variables, such as, perceived health, leisure and income, as these were pointed out as significant for this group of older adults for promoting adjustment to aging in late adulthood. 1.von Humboldt S et al. How do older adults experience intergenerational relationships? Different cultures, ambivalent feelings. Educ.Gerontol.2018;44(8):501-513. 2.von Humboldt S et al. Analyzing adjustment to aging and subjective age from Angolan and Portuguese community-dwelling older adults' perspectives. Int.J.Gerontol.2013;7(4):209-215. 3.von Humboldt S et al. What influences the subjective wellbeing of older adults?: A systematic review of the literature. Rev.Argent.Clín.Psicol.2014;23(3):219-230.

